# The novel strategies for next-generation cancer treatment: miRNA combined with chemotherapeutic agents for the treatment of cancer

**DOI:** 10.18632/oncotarget.24309

**Published:** 2018-01-23

**Authors:** Chiranjib Chakraborty, Ashish Ranjan Sharma, Garima Sharma, Bimal Kumar Sarkar, Sang-Soo Lee

**Affiliations:** ^1^ Institute for Skeletal Aging and Orthopedic Surgery, Hallym University-Chuncheon Sacred Heart Hospital, Chuncheon 24252, Republic of Korea; ^2^ Department of Bioinformatics, School of Computer Sciences, Galgotias University, Greater Noida 203201, Uttar Pradesh, India; ^3^ Department of Physics, School of Basic and Applied Science, Galgotias University, Greater Noida 203201, Uttar Pradesh, India

**Keywords:** miRNA, combination therapy, chemotherapeutic agents, cancer, next-generation therapy

## Abstract

Medical practitioners are recommending combination therapy in cancer for its various advantages. Combination therapy increases the efficacy of treatment due to its synergistic effects in cancer treatment. In this post-genomic era, microRNAs (miRNAs) are receiving attention for their role in human disease and disease therapy. In this review, we discuss the combination of miRNAs and chemotherapeutic agents for cancer treatment. Moreover, we attempted to portray the role of miRNAs in cancer therapy; outline combination therapy, especially chemo-combination therapy, and discuss the basis for miRNA-based chemo-combination therapies and chemo-combination therapy with miRNA for cancer treatment.

## INTRODUCTION

Since human genome sequencing and the advancement of the post-genomic era, a new wave of RNomics has been generated, and our understanding about small non-coding RNAs (ncRNAs) has grown exponentially [1, 2]. Among these ncRNAs, microRNAs (miRNAs) are receiving increasing attention because they regulate gene expression at the post-transcriptional level. miRNAs are non-protein-encoding RNAs that range in size from 19 to 25 nucleotides (nt). As for usual protein-coding mRNA, it has been noted that miRNAs are transcribed through RNA polymerase II. Then, they are spliced, capped and poly-adenylated, resulting in the primitive miRNA (pri-miRNA). Subsequently pri-miRNAs are processed through an endonuclease (RNase III endonuclease, called Drosha), generating the hairpin ‘precursor’ miRNAs. In animals, these ‘precursor’ miRNAs are ∼70 nt long in the nucleus. However, in plants, the precursor miRNA length is longer at approximately ∼100 nt in the nucleus [3]. Then, the hairpin precursor miRNAs bind to exportin-5 and RNA GTPase, which permits the transport of the molecules into the cytoplasm. The “precursor” miRNA is then processed into ∼22 nt miRNA duplexes by Dicer [4]. miRNAs participate in several cellular functions, such as cell differentiation, cell cycle regulation and apoptosis [5]. miRNAs are important molecules with a vital role in human diseases and manages different cellular pathways in human diseases [6–8]. Chakraborty et al., described the occurrence of miRNAs that were linked with the regulation of key proteins related to insulin resistance and the insulin signaling pathway [9]. In another study, Chakraborty et al., described the occurrence and responsibility of miRNAs in the insulin signaling pathway and associated pancreatic cancer development [10]. The role of miRNAs in controlling MAPK signaling pathways in chronic myeloid leukemia has recently been reviewed [11]. Chakraborty et al., also reviewed miRNA controlled cancer stem cells that can help to understand the role of miRNA in carcinogenesis [12]. Sharma et al., described the role of miRNAs in cytokine signaling pathways associated with rheumatoid arthritis [13]. Alternatively, it has been anticipated that chemotherapeutic agents may perform their anticancer function activity through modulating miRNA expression [14].

It has been noted that miRNAs genes are located in specific key sites. For example, cancer-associated delicate sites, cancer-associated genomic regions or areas those are associated with individual subsets of solid tumors and hematological malignancies [15, 16]. The miRNAs can work as tumor suppressors, such as if the miRNA, which up-regulates malignancies, suppresses the malignant development function in a normal cell [17]. Several recent discoveries have shown that miRNAs have potential applications in cancer treatment. Moreover, studies advocate the possibility of restoring tumor suppressive miRNAs and targeting oncogenic miRNAs for cancer therapy [18, 19].

Worldwide, cancer is an important cause of death in both economically developed countries as well as in developing countries. According to the WHO (World Health Organization), approximately 8.2 million deaths occurred by cancer in 2012, throughout the globe. It was estimated that 14.1 million new cancer cases must have occurred in 2012, worldwide [20]. At present, cancer is the top cause of death in 21 states in USA [21]. It is also the prime cause of death in Asiatic countries and Europe. Among all cancers, breast, lung, pancreatic, liver, stomach, colon and prostate cancer are the leading cause of cancer deaths around the world. It has been reported that the death rates are also increasing for cancers of the pancreas, liver, and uterine corpus in the USA. Among all cancer related deaths recorded in 2012, lung cancer is a foremost cause of death worldwide, accounting for approximately 1.59 million deaths. Other important cancer deaths are liver cancer deaths (about 745,000); stomach cancer deaths (about 723,000) and breast cancer deaths (about 521,000) [22]. At present, various therapeutic strategies for cancer management are available and there are three main types, chemotherapy, surgery and radiotherapy. However, chemotherapy plays a significant role in cancer treatment. Conversely, several advances in the field of surgery and radiotherapy have been recorded over time [23].

At present, chemotherapy remains the primary treatment for cancer, and it is frequently used to treat patients with metastases. It is first line of treatment against cancer, both curable and advanced cancers, and it improves the general survival and quality of life for cancer patients. The age of chemotherapy started with its use during the 1940s using nitrogen mustards and antifolate drugs [24]. However, chemotherapy is associated with several side effects usually caused by adverse effect of the anti-cancerous drugs on blood forming cells in the bone marrow, hair follicles and cells in the digestive tract and reproductive system. Hence, currently, there is an increasing interest in the combination/conjugation of chemotherapy to avoid this painful side effect. The combination/conjugation chemotherapy focuses on maximizing the efficacy and minimizing systemic toxicity. Finally, combination/conjugation chemotherapy aims to decrease the drug doses [25, 26].

In this article, we evaluated the combination of miRNAs and chemotherapeutic agents for treating cancer. We also discussed the role of miRNAs in cancer therapy, provided an overview of combination therapy of chemotherapeutic agents, described the reason behind miRNA-based chemo-combination therapies, and detailed the use of combination therapy of miRNA with chemotherapeutic agents for cancer therapy.

### miRNAs and cancer therapy

Several new strategies have been added in the last 5 years, which can increase survival rate and reduces mortality rate; miRNA therapy is one of these strategies [27, 28]. There are two miRNA-based treatment approaches for cancer, which are miRNA reduction or inhibition therapy and miRNA replacement or restoration therapy for cancer.

### miRNA replacement or restoration therapy

miRNA replacement or restoration therapy employs the reuse of miRNAs that are deleted or downregulated in cancers [29, 30]. Some studies have been performed on this topic. Bonci et al., have introduced miRNA in prostate cancer cell lines. They have used miR-15a and miR-16-1 clusters in prostate cancer cell lines where they have induced apoptosis, blocked proliferation and controlled prostate cancer by targeting numerous oncogenic activities [31]. Trang et al., performed systemic delivery of tumor suppressor miRNAs using miR-34a and let-7. This approach decreased the tumor load in a KRAS (K-ras; a proto-oncogene)-activated non-small cell lung cancer mouse model. The miR-34a accumulated in the tumor tissue, which was followed by down regulation of its direct targets. This finally caused a 60% reduction in the tumor area [32]. Here, researchers have used the lipid-based delivery vehicle whereby, miRNA (miR-34a or let-7 mimic) formed a complex with a lipid suspension. In another study, Tazawa et al., performed systemic delivery of miR-34a and miR-16 and successfully repressed the development of prostate and colon cancer [33]. Researchers have used adenovirus expression of let-7a in a KRAS mutant mouse model. In these studies, researchers efficiently controlled the growth of lung cancer in a xenograft mouse model or murine lung tumor model [32, 34].

The first commercial miRNA restoration therapy is MRX34. This MRX34 is a liposome-based miR-34 mimic that can be intravenously injected. It is currently being investigated in Phase I clinical trials for advanced hepatocellular carcinoma patients [35].

### miRNA reduction or inhibition therapy

miRNA reduction or inhibition therapy can inactivate those miRNAs that are overexpressed or upregulated in cancers, especially in tumors [28]. Several miRNA inhibitory agents have been studied over time. Some are locked nucleic acid or LNA [36], antisense anti-miR oligonucleotides [37], small molecule inhibitors of miRNAs [38] and miRNA sponges [39, 40].

LNA is a nucleic acid analogue. LNA may consist of one or more nucleotide monomers. Anti-miRNA oligonucleotides (AMOs) (2’-O-methyl AMOs and 2’-O-methoxyethyl) are LNA anti-miRs. In this case of LNA, there is an additional methylene bridge linking the 2′-O and 4′-C atoms. This ‘locks’ the ribose ring. In this particular region, a C3’-endo or C2’-endo conformation can be observed [38]. There may be a high-affinity of Watson-Crick binding to target mRNAs in the case of LNA. Elmén et al., performed LNA-mediated miRNA silencing in non-human primates. They provided LNA-anti-miR intravenous injections in primate models i.e., African green monkeys, which caused LNA-anti-miR uptake in the cytoplasm. It formed a configuration of a heteroduplexes, connecting LNA-anti-miR and miR-122 [41]. Sharifi and Salehi blocked miR-92a-3p with LNA-anti-miR92a-3p.This LNA inhibition encouraged apoptosis and stop cell propagation in human acute leukemia [42]. Commercial LNA-anti-miR has entered into a clinical trial run by the Santaris Pharma. Miravirsen, an LNA-anti-miR-122, has efficiency that was estimated in Phases I and IIa clinical trials. This LNA-anti-miR was used to manage hepatitis C virus (HCV) [43].

Several small molecules are reported to inhibit miRNAs. In 2008, Gumi reddy et al., reported on small-molecule inhibitors (SMIRs) of miR-21. In this study, a luciferase-based reporter assay was also developed to recognize large-scale drug screening in which possible miRNA-specific small molecule inhibitors were used [44]. Naro et al., reported on a new type of small-molecule inhibitors, such as aryl amide small-molecule inhibitors of miR-21. This miRNA was over expressed in different types of human cancers and HeLa cells [45]. miRNA sponges antagonize miRNA, which has RNA transcripts with multiple tandem repeats. It has been noted that sponge RNAs enclose binding sites opposite to a miRNA [39]. Liang et al., developed long non-coding RNA (lncRNA), such as lncRNA H19 that act as miRNA sponges in colorectal cancer [46]. Ma et al., developed a miRNA sponge that can hinder miR-9 in extremely malignant cells. This sponge is used in the pulmonary micro-metastasis in murine models, which slows metastasis development. miR-9 intensity is linked to MYCN amplification, metastatic status and tumor ranking [47].

### Difficulties with miRNA therapeutics

There are some challenges in developing miRNA-based therapeutics. Low RNA stability has been noted *in vivo*. The half-life of RNA increases with miRNA therapy [48]. Another complexity is tumor-specific delivery and retention of miRNAs. miRNAs target numerous mRNAs, which is also a challenge. Sometimes, targeted delivery molecules could be stuck in first-pass metabolism with speedy localization of small molecules delivered to organs, such as the liver and kidneys [49]. However, nanoparticle delivery can resolve the problem. Nanoparticles that range from 15 to 100 nm are the best size for systemic delivery [50], which facilitates target-specific delivery of miRNA.

### Overview of ‘combination therapy’ with chemotherapeutic agents

‘Combination therapy’ is used in cancer treatment. The first successful combination chemotherapy could cure acute childhood leukemia and was then extended to treat Hodgkin’s disease and cure advanced cancers [51]. To treat acute childhood leukemia, a POMP chemotherapy regimen was used. Conversely, to treat lymphoma, MOPP chemotherapy regimen was used. Furthermore, rational drug combinations can help interfere with multiple tumor cell survival pathways, offering great promise in reducing tumor metastasis [52].

Since the use of drug combinations, the national occurrence and mortality of cancer began to decline in 1990. Cancer mortality has continued to decline annually in spite of the aging U.S. population [53, 54]. The rate has further declined [55] because of advances in cancer treatment [24].

Despite considerable success with ‘combination therapy’ in chemotherapy treatment programs, resistance to chemotherapeutic treatment has emerged as a challenge to effective treatment [56, 57]. It has been noted that many cancer types are susceptible to chemotherapy; over time, cancer cells can develop resistance through different mechanisms, such as DNA mutations and metabolic changes that inhibit and degrade drugs [62]. In this post-genomic era, current genomic and proteomic research have shown novel insights into molecular mechanisms of cancer chemo-sensitivity and chemo-resistance [58, 59]. However, vast evidence has revealed that drug resistance is the major basis of treatment failure.

Presently, the concept of ‘combination therapy’ with various miRNAs together with chemotherapeutic agents may help cancer patients by providing synergistic effects.

One such type of ‘combination therapy’ for treating HCV is being evaluated in a Phase II clinical trial. In this case, one miRNA is anti-miR (Brand name: miravirsen), which is used with antiviral drugs, such as telaprevir and ribavirin, to treat HCV [43, 60].

### Rationale for the miRNA based chemo-combination therapies

**Figure 1:**
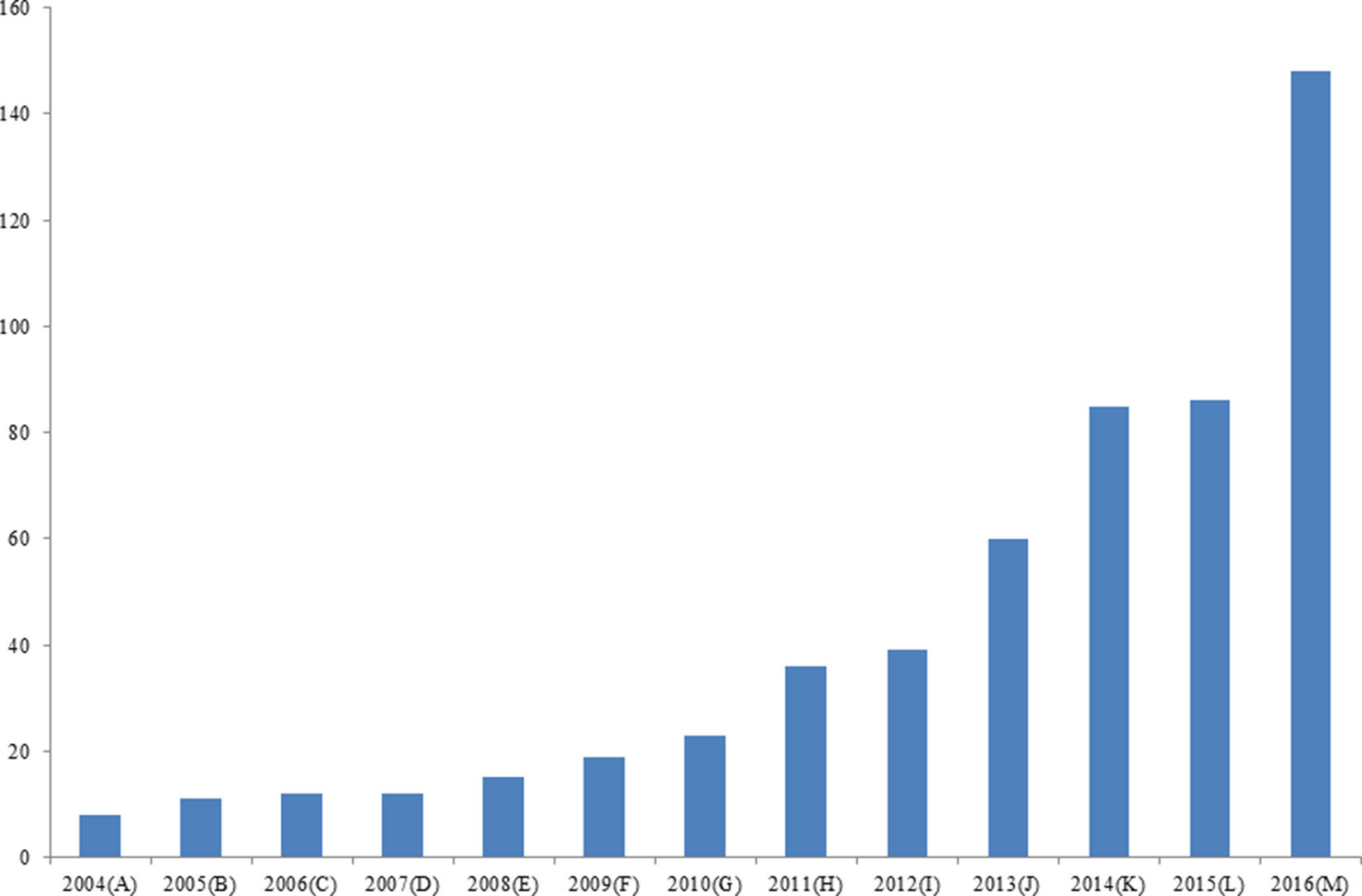
Increasing trend in the publication for “chemo-resistance” research Keyword search was performed from PUBMED, NCBI database.

**Figure 2:**
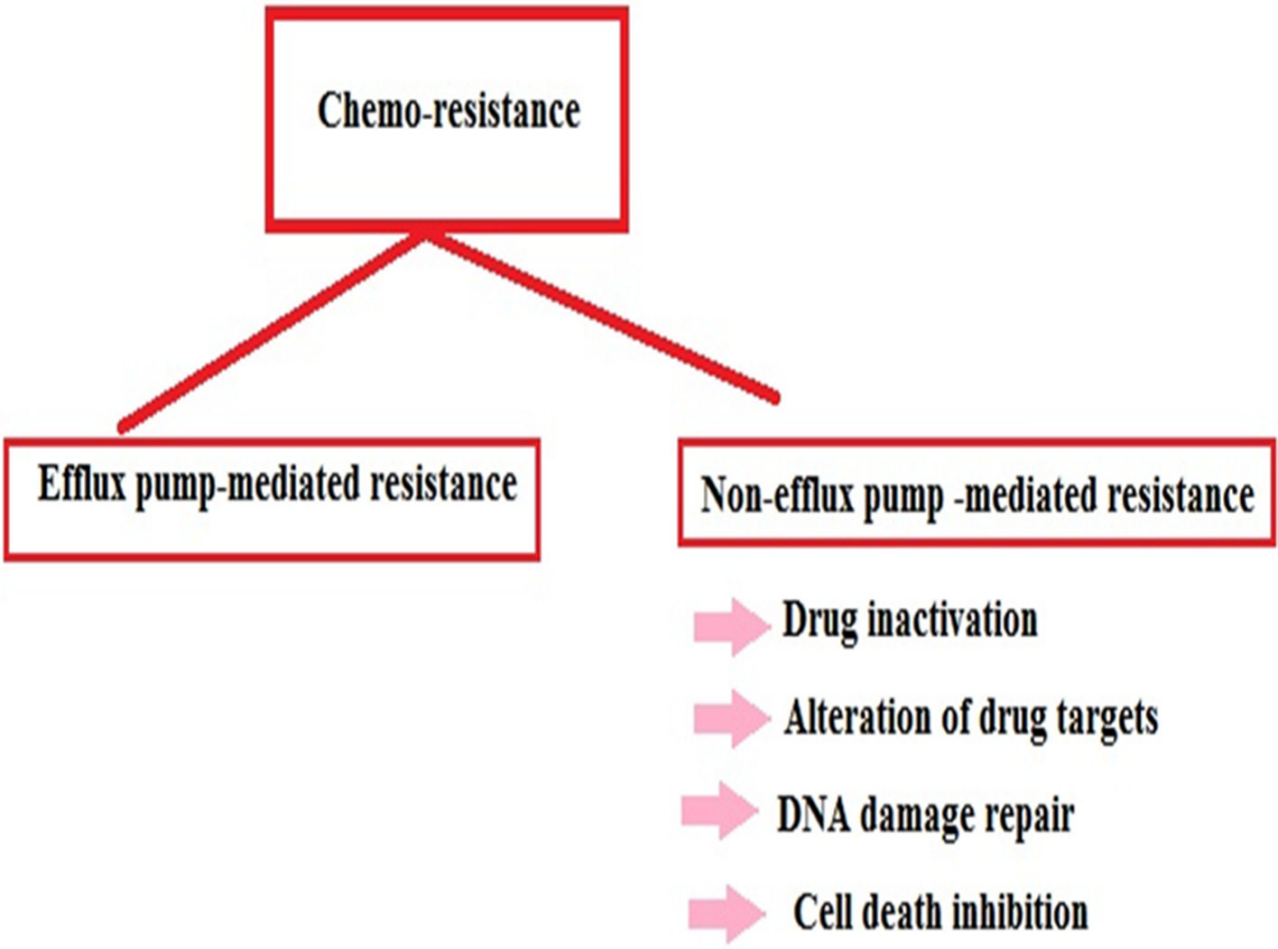
The chart describes different methods of chemo-resistance

There is resistance against chemotherapeutic drugs and chemotherapeutic resistance reports are increasing day by day (Figure 1). It has been suggests that chemo-resistant cancer cells that continue to be active after the chemotherapy are responsible for tumor recurrence [61, 62]. However, we need to understand the mechanisms by which cancer cells develop chemo-resistance. Some key mechanisms have been suggested by which the tumor cells are more likely to develop chemo/drug resistance; these include efflux pump-mediated resistance in chemotherapy and non-efflux pump-mediated resistance in chemotherapy (Figure 2).

### Efflux pump-mediated resistance in chemotherapy

Efflux pumps are present in all cells and they guard the cells from chemical toxicity, especially from organic chemical toxicity. It is an energy-dependent drug efflux pump in prokaryotic and eukaryotic cells [62, 63]. However, in cancer cells, it has been observed that the MDR-1 gene is mainly accountable for triggering the efflux pump. Similarly, some other genes are also involved in triggering the efflux pump, such as MDR-1a and MDR1b. P-glycoprotein is connected to the MDR1 gene [64]. When this gene is over-expressed in cancer cells, it contributes to cancer chemotherapy resistance. Cytotoxicity results from inhibition of cytochrome P-450 enzymes, augmenting the plasma concentration of anticancer agents [65]. Finally, efflux pumps get repressed.

The ATP-binding cassette (ABC) transporter family protein members enable efflux pumps and are present in the plasma membranes of healthy cells. The ABC transporter is part of a normal physiological process. P-glycoprotein efflux pumps are one of the first members of the ABC transporter family protein.

The P-glycoprotein-related efflux pumps are usually present on the cell membrane, and they may be found in the nuclear membrane. These P-glycoprotein-related efflux pumps have the capacity to attach to molecules that are positive or neutrally charged [66]. Researchers have reported that most chemotherapeutic drugs are impartial or either positively charged under cellular pH. Therefore, chemotherapeutic drugs act as a substrate for P-glycoprotein-related efflux pumps, and chemotherapeutic drugs can be pumped out of the cell [67].

### Non-efflux pump-mediated resistance in chemotherapy

Non-efflux pump-mediated resistance in chemotherapy is related to drug inactivation, alteration of drug targets, DNA damage repair, cell death inhibition, etc. Housman et al., eloquently described drug inactivation, alteration of drug targets, DNA damage repair, cell death inhibition, etc. [65].

Rueff and Rodrigues described cancer drug resistance from a genetic point of view. They described distorted levels of miRNAs that may act as upstream or downstream effectors [68]. The same researchers have also reported on the mutation of drug targets in targeted therapy as well as modification in the checkpoints, cell cycle, and tumor micro-environment.

### Combination therapy of mirna with chemotherapeutic agents for cancer therapy

Use of different ‘combination therapy’ with several types of miRNA along with the various type of chemotherapeutic agents for the treatment of cancer have been reported (Table 1).

**Table 1:** Different miRNA combine with chemotherapeutic agents used for ‘combination therapy’to treat the different cancers

Sl.No	Different miRNA	‘Combination therapy’ with chemotherapeutic agents	Treatment for different cancers/cancer cells	Reference
1.	miR-205	Gemcitabine	This ‘combination therapy’ used to treat the pancreatic cancer. It inhibited of tumor growth in gemcitabine resistant pancreatic cancer cells (MIA PaCa-2(R) and CAPAN-1(R) cells).	[71]
2.	miR-34a	Paclitaxel	This ‘combination therapy’ was used to treat cancers where miR-34a was integrated jointly with paclitaxel into solid lipid nanoparticles (miSLNs-34a/PTX).	[73]
3.	miR-34a	Doxorubicin	This ‘combination therapy’ was inhibited prostate cancer metastasis and progenitor cells. It hindered prostate cancer metastasis through repressing CD44.	[75]
4.	miR-129	Fluorouracil (5-FU)	This ‘combination therapy’ was used to treat colorectal tumor mouse model.	[79]
5.	miR-145	Fluorouracil (5-FU)	This ‘combination therapy’ was used to treat both breast cancer cells as well as the breast cancer mouse model.	[80]
6.	miR-34a	Docetaxel	This ‘combination therapy’ was used to treat metastatic breast cancer.	[85]

Gemcitabine is a novel anticancer nucleoside that is a deoxycytidine analog [69, 70]. The clinical efficiency of gemcitabine has slowed in cancer because of chemo-resistance and rapid plasma metabolism. Tumor suppressor miRNA-205 has been used in combination with gemcitabine to treat pancreatic cancer. In this model, gemcitabine-conjugated cationic copolymer delivery was performed with miR-205. Significant inhibition of tumor growth was noted in gemcitabine-resistant pancreatic cancer cells (MIA PaCa-2(R) and CAPAN-1(R) cells) [71].

Paclitaxel (PTX) is also a novel anticancer agent that is used to treat different cancer types. The brand name is Taxol, which is given as an intravenous therapy [72]. Shi et al., developed a co-delivery system using miR-34a and paclitaxel and evaluated the synergistic effects of this combination therapy for cancer treatment. In this system, miR-34a was jointly integrated with paclitaxel in solid lipid nanoparticles (miSLNs-34a/PTX), which are cationic. This co-delivery system improved the anti-cancer activity in mice, and it has substantial potential to inhibit tumor growth and remove cancer cell populations [73].

Doxorubicin (DOX) is a chemotherapeutic medicine that is used to treat different cancer types. One of its trade names is Adriamycin [74]. One miRNA, such as miR-34a, has anticancer activity. It has been found that delivery of miR-34a inhibits prostate cancer metastasis and progenitor cells. It potently hinders prostate cancer metastasis through repressing CD44 [75].

Using hyaluronic acid-chitosan nanoparticles, Deng et al., performed co-delivery of miR-34a and DOX, which were proficiently capsulated into nanoparticles and delivered to tumor cells or tumor tissues. In this delivery system, DOX-miR-34a co-loaded hyaluronic-chitosan nanoparticles were effectively arranged through the ionotropic gelation method. The combination drug (DOX-miR-34a) was shown to improve the anti-tumor activity by restraining the expression of an anti-apoptosis gene, Bcl-2 [76].

Fluorouracil (5-FU) is also a novel anticancer agent used to treat different cancer types. The brand name is Adrucil, which is given as intravenous therapy [77]. 5-FU resistance is occasionally noted, which is the major reason for cancer management failure [78]. MiR-129 has anticancer miRNA activity, activating apoptosis by suppressing a key anti-apoptotic protein, B-cell lymphoma-2 (Bcl-2). Karaayvaz et al., reported that this miRNA augments 5-FU cytotoxicity in a colorectal tumor mouse model [79]. In another work by Kim et al., an adenoviral constructed miR-145 was developed which was used to treat the breast cancer cells *in vitro*. On the other hand, same compound (adenoviral constructed miR-145 or Ad-miR-145) was used to treat the orthotopic mouse breast cancer model *in vivo*. It was found that the adenoviral constructed miR-145 suppressed the cell growth and motility in both breast cancer cells as well in the breast cancer mouse model. This adenoviral constructed miR-145 was used in combination with 5-fluorouracil which considerably enhanced the antitumor effects in relation to 5-fluorouracil treatment alone [80, 81].

Docetaxel (DTX) is a unique broad-spectrum chemotherapeutic agent. It is used to treat different cancer types especially, solid cancers [82, 83]. Hermeking (2010) described that miRNA-34a is a powerful tumor suppressor [84]. Recently nanocarrier was used to deliver both the molecule of DTX and miRNA-34a to treat metastatic breast cancer. In this case, a core-shell of nanocarrier was prepared which was coated by cationic albumin to deliver the combination of therapeutic molecules. It was observed that the co-delivery of both the molecules (docetaxel and miRNA-34a) can efficiently treat the metastatic breast cancer compare to docetaxel or miRNA-34a alone [85].

### Concluding remarks and future perspectives

**Figure 3:**
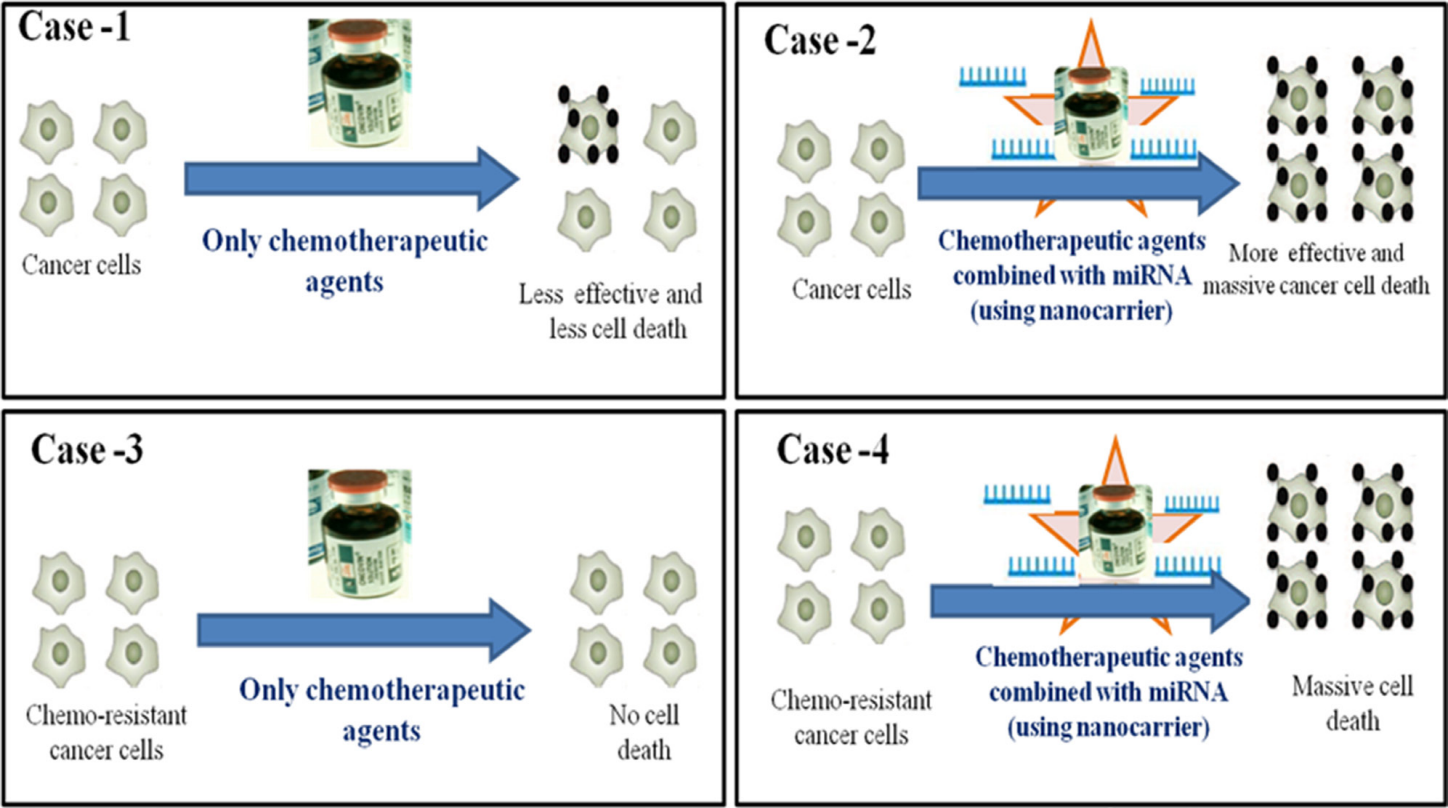
Role of combination therapy of miRNA and chemotherapeutic agent to the cancer cells Schematic diagram depicting the outcomes of various case studies related to the treatment of chemotherapeutic agent alone or in combination with miRNA. Case-1: Cancer cells treated only with the chemotherapeutic agents induces less effective cancer cell death than when treated along with chemotherapeutic agents (using nanocarrier) Case-2. Case-3: Chemo-resistant cancer cells treated only with the chemotherapeutic agents demonstrate no cells death due to chemo-resistance. Case-4: While Chemo-resistant cancer cells when treated with chemotherapeutic agents combined with miRNA (using nanocarrier) causes massive cancer cells death.

**Figure 4:**
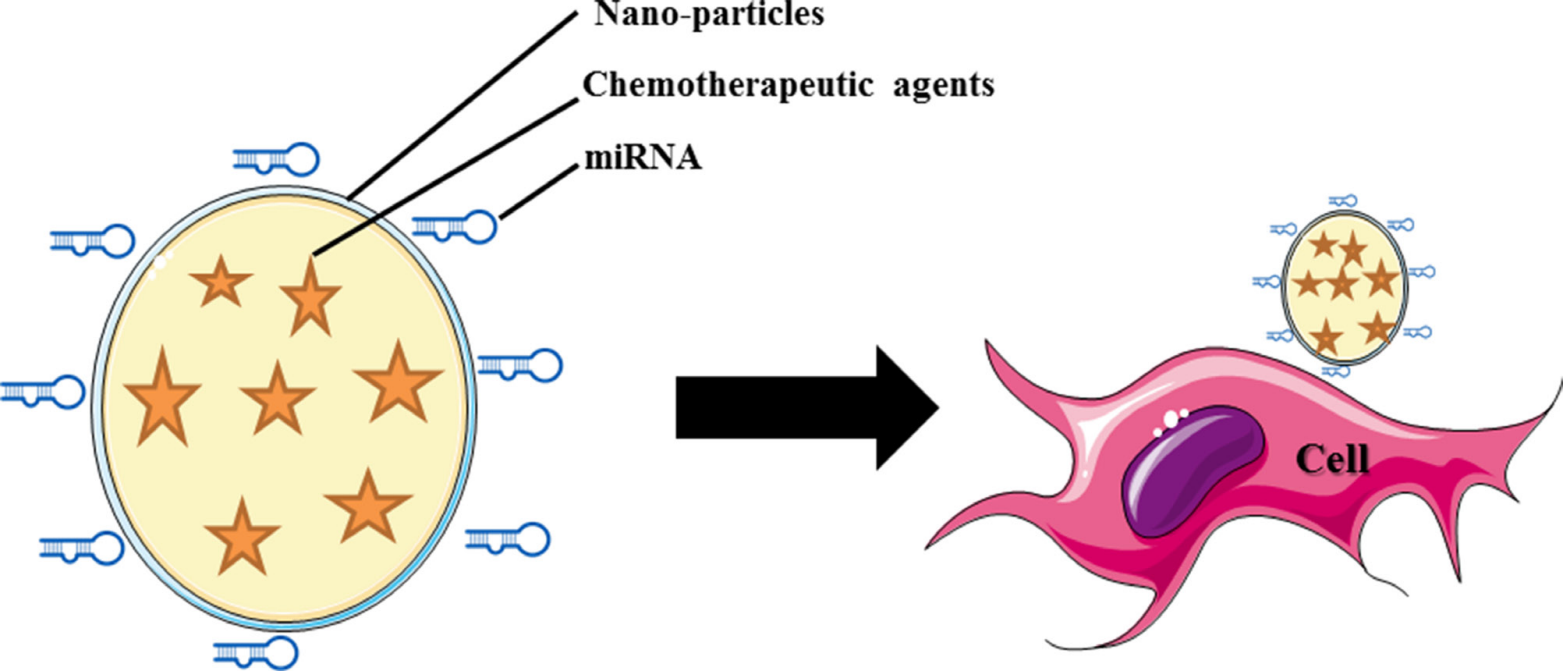
Schematic illustration of the nanoparticle based systemic delivery of miRNA and chemotherapeutic agents to the cell

The combination of miRNA and chemotherapeutic agents provides hope as an alternative that can be used to overcome chemo resistance in cancer treatment (Figure 3). Gathering information on clinical trials using a combined delivery consisting of miRNA and anticancer agents is very challenging. However, pharmacokinetics and pharmacodynamics data on the combination of miRNA and chemotherapeutic agents are needed. Studies on the safety of miRNA combined with chemotherapeutic agents will be beneficial in the future, especially a study on the direction of toxicity and the immune response. Different delivery systems, including a nanoparticle delivery system, will help in the delivery of these combined chemotherapeutic agents (Figure 4) [86]. Advancement of novel nanocarriers and different drug delivery technologies will facilitate the delivery of combination miRNA and chemotherapeutic treatments. However, there are challenges with the combination of miRNA and chemotherapy, and there is significant room for developing miRNA-conjugated chemotherapeutic agents. The era of combining miRNA with chemotherapy is emerging and will expand in the next few decades.

## Abbreviations

POMP: procarbazine, vincristine (Oncovin), nitrogen mustard (mustine) and prednisone
MOPP: nitrogen mustard, vincristine, prednisone and procarbazine
MAPK: Mitogen-activated protein kinase
MDR: Multidrug resistance
